# FDG-PET underscores the key role of the thalamus in frontotemporal lobar degeneration caused by C9ORF72 mutations

**DOI:** 10.1038/s41398-019-0381-1

**Published:** 2019-01-31

**Authors:** Janine Diehl-Schmid, Abigail Licata, Oliver Goldhardt, Hans Förstl, Igor Yakushew, Markus Otto, Sarah Anderl-Straub, Ambros Beer, Albert Christian Ludolph, Georg Bernhard Landwehrmeyer, Johannes Levin, Adrian Danek, Klaus Fliessbach, Annika Spottke, Klaus Fassbender, Epameinondas Lyros, Johannes Prudlo, Bernd Joachim Krause, Alexander Volk, Dieter Edbauer, Matthias Leopold Schroeter, Alexander Drzezga, Johannes Kornhuber, Martin Lauer, FTLDc Study Group, Nibal Ackl, Christine v. Arnim, Joachim Brumberg, Florian Gärtner, Holger Jahn, Elisabeth Kasper, Jan Kassubek, Catharina Prix, Lina Riedl, Carola Roßmeier, Sonja Schönecker, Elisa Semler, Stefan Teipel, Christine Westerteicher, Elisabeth Wlasich, Timo Grimmer

**Affiliations:** 1 Department of Psychiatry, Klinikum rechts der Isar, Technical University of Munich, Munich, Germany; 2 Department of Nuclear Medicine, Klinikum rechts der Isar, Technical University of Munich, Munich, Germany; 3 Department of Neurology, Ulm University Hospital, Ulm, Germany; 4 Neurologische Klinik, Ludwig-Maximilians-Universität München, Munich, Germany; 5 German Center for Neurodegenerative Diseases (DZNE), Site Munich, Munich, Germany; 6 Department of Neurodegenerative Diseases and Geriatric Psychiatry, University of Bonn, Bonn, Germany; 7 German Center for Neurodegenerative Diseases (DZNE), Site Bonn, Bonn, Germany; 8 Department of Neurology, University of Bonn, Bonn, Germany; 9 Department of Neurology, Saarland University, Homburg/Saar, Germany; 10 Department of Neurology, Rostock University Medical Center, Rostock, Germany; 11 Department of Nuclear Medicine, Rostock University Medical Center, Rostock, Germany; 12 Institute of Human Genetics, University Medical Centre Hamburg-Eppendorf, Hamburg, Germany; 13 Munich Cluster for System Neurology (SyNergy), Munich, Germany; 14 Clinic for Cognitive Neurology, University Clinic Leipzig, Leipzig, Germany; 15 Max Planck Institute for Human Cognitive and Brain Sciences, Leipzig, Germany; 16 Department of Nuclear Medicine, University of Cologne, Cologne, Germany; 17 German Center for Neurodegenerative Diseases (DZNE), Site Cologne, Cologne, Germany; 18 Department of Psychiatry and Psychotherapy, Friedrich-Alexander-University of Erlangen-Nuremberg, Erlangen, Germany; 19 Department of Psychiatry, Psychosomatics and Psychotherapy, University Hospital of Würzburg, Würzburg, Germany; 20 Ludwig-Maximilians-Universität München, Munich, Germany; 21 Department of Nuclear Medicine, University Hospital and Julius-Maximilians-University, Würzburg, Germany; 22 Department of Nuclear Medicine, University of Bonn, Bonn, Germany; 23 Department of Psychiatry and Psychotherapy, University Medical Center Hamburg-Eppendorf, Hamburg, Germany; 24 BG Klinikum Bergmannstrost, Halle/Saale, Germany; 25 Department of Psychosomatic Medicine, Rostock University Medical Center, Rostock, Germany; 26 German Center for Neurodegenerative Diseases (DZNE), Site Rostock, Rostock, Germany

## Abstract

C9ORF72 mutations are the most common cause of familial frontotemporal lobar degeneration (FTLD) and amyotrophic lateral sclerosis (ALS). MRI studies have investigated structural changes in C9ORF72-associated FTLD (C9FTLD) and provided first insights about a prominent involvement of the thalamus and the cerebellum. Our multicenter, ^18^F-fluorodeoxyglucose positron-emission tomography study of 22 mutation carriers with FTLD, 22 matched non-carriers with FTLD, and 23 cognitively healthy controls provided valuable insights into functional changes in C9FTLD: compared to non-carriers, mutation carriers showed a significant reduction of glucose metabolism in both thalami, underscoring the key role of the thalamus in C9FTLD. Thalamic metabolism did not correlate with disease severity, duration of disease, or the presence of psychotic symptoms. Against our expectations we could not demonstrate a cerebellar hypometabolism in carriers or non-carriers. Future imaging and neuropathological studies in large patient cohorts are required to further elucidate the central role of the thalamus in C9FTLD.

## Introduction

In 2011 a hexanucleotide repeat expansion mutation in a non-coding region of the *C9ORF72* gene was discovered^1,2^, which appeared to be the most common cause of familial frontotemporal lobar degeneration (FTLD) and amyotrophic lateral sclerosis (ALS).

The suggested disease mechanisms are a loss of function through a reduced expression of C9ORF72 protein, the formation of both nuclear foci of the sense and antisense repeat RNA, and non-ATG mediated sense and antisense translation of the expansion itself. Repeat translation leads to formation and cellular accumulation of five different dipeptide repeat proteins (DPRs), which show differential toxicity in various model systems (for an overview see ref. ^3^). DPRs are a cardinal pathological feature of expansion carriers. They are most widespread throughout the neocortex, hippocampus, thalamus, and cerebellum, and rare in the spinal cord including motor neurons^4^. Thus, DPR distribution is spatially not correlated with the areas of most prominent neurodegeneration in ALS and FTLD cases.

Additionally, FTLD cases with the C9ORF72 mutation (C9FTLD) are pathologically characterized by inclusion bodies within neurons and glial cells that contain transactive response DNA-binding protein (TDP-43) and accumulate in the frontal and temporal cortex, hippocampus, and pyramidal motor system, as well as in a wide range of other neuroanatomical regions including the amygdala, striatum, and thalamus. TDP-43 pathology is not exclusively a pathological feature of C9ORF72 expansion carriers but is a major pathological protein also in sporadic ALS and the majority of familial and sporadic, tau-negative cases. In contrast to DPR pathology, the distribution of TDP-43 pathology appears to be highly correlated with areas of neurodegeneration and clinical symptoms (for an overview see ref. ^5^).

In the last years, detailed series of C9ORF72 mutation carriers have been reported and the clinical features have been described. Several studies detected a higher prevalence of psychiatric presentations and psychotic symptoms in carriers with behavioral variant frontotemporal dementia (bvFTD; C9FTD) as compared to non-carriers^6,7^. Typical features include delusions and (in some cases odd somatoform or tactile) hallucinations^8^.

A wealth of structural neuroimaging studies has described the typical brain changes in C9FTLD. Various cohort studies using voxel-based morphometry have shown atrophy distributed in frontal, insular, temporal, and parietal cortical as well as subcortical regions. In accordance with neuropathological studies^9,10^ thalamic and cerebellar involvement is relatively prominent in C9FTLD, which has been suggested to constitute a group-level signature of C9ORF72 expansions^11^. This conclusion is supported by longitudinal studies that show a preferential volume loss most consistently in the thalamus and the cerebellum in symptomatic patients with C9FTLD^12^. A recent magnetic resonance imaging (MRI) study in asymptomatic carriers of the C9ORF72 expansion has shown that subcortical areas including thalamus, insula, and posterior cortical areas differed between carriers and controls at 25 years before expected onset, followed by later involvement of the frontal and temporal lobes and the cerebellum^11^. One study found that in C9FTD and C9ALS the salience network connectivity reduction correlated with atrophy in the left medial pulvinar thalamic nucleus, and that this region further showed diminished connectivity to key salience hubs^13^.

So far, only a few case reports and two studies of ALS patients with C9ORF72 mutation have described the results of cranial ^18^F-fluorodeoxyglucose positron-emission tomography (FDG-PET). A smaller study revealed that C9ALS patients have lower glucose metabolism than ALS patients without mutation, particularly in the limbic system, caudate nucleus, and thalamus^14^.

FDG-PET studies in FTLD with C9ORF72 mutation have not yet been performed.

Hence, the aim of our study was to investigate the cerebral glucose metabolism as measured with FDG-PET in FTLD-patients with and without C9ORF72 mutations (C9+ and C9−).

Using voxel-based comparisons we aimed to compare the cerebral glucose metabolism between patients with and without mutation and cognitively healthy controls (HCs). Furthermore, we aimed to investigate, if thalamic and cerebellar glucose uptake is reduced in C9+ as compared to C9− using region-of-interest (ROI) analyses, and if the thalamic or the cerebellar metabolism correlates with clinical features. In particular, since imaging studies in schizophrenia patients had shown a correlation of positive symptoms (i.e. hallucinations and delusions) with thalamic volumes^15^, we aimed to explore if reduced thalamic uptake corresponds with the presence of psychotic symptoms in our patient cohort.

## Subjects and methods

### Subjects

Patients were recruited from different clinical sites (Technical University of Munich, University of Ulm, Ludwig-Maximilians-Universität of Munich, University of Bonn, University of Rostock, and University of Homburg) coordinated by the German FTLD consortium (FTLDc, www.ftld.de), a quality-controlled, monitored multicenter initiative to register and trace patients with FTLD spectrum disorders^16^. The study was approved by the local ethics committees of all FTLDc centers (central ethics committee at University of Ulm: # 39/11, 8 March 2011). Each patient, participant, caregiver, or legal representative provided written informed consent for the study according to institutional guidelines. The study was conducted according to the principles expressed in the Declaration of Helsinki.

Of the 650 patients with FTLD spectrum disorders included between April 2011 and December 2016 into the FTLDc, 520 were screened for C9ORF72 mutations. In 38 patients a mutation was detected. The mutation carriers who had undergone a cerebral FDG-PET scan at study inclusion (*n* = 22) were included in the present study. A non-mutation carrier from the FTLDc was matched to every mutation carrier. Matching criteria applied were: subdiagnosis, gender, age, dementia severity (as measured by Clinical Dementia Rating (CDR)^17^), and FTLDc study site. In all patients a comprehensive assessment according to the FTLDc protocol was obtained, including neurological and psychiatric examination, routine laboratory, neuroimaging with MRI and/or FDG-PET. A detailed neuropsychological examination was performed, including the German version of the Consortium to Establish a Registry of Alzheimer’s Disease-Neuropsychological Assessment Battery (CERAD-NAB)^18^, which contains the Mini-Mental State Examination (MMSE)^19^. Dementia severity was measured using the CDR scale^17^. In 18 patients (C9+ and C9−, 9 each) cerebrospinal fluid biomarkers (total-tau, phospho-tau, Abeta 1-42, and neurofilament light chains) were available. From 10 patients (3 mutation carriers and 7 non-carriers) genotyping for GRN and MAPT had been performed, which excluded mutations in all patients.

### Healthy controls

Images were compared to those of a group of 23 age- and gender-matched HC subjects without subjective or objective cognitive impairment (9 male/13 female; age: 64.78 + 6.13 years at PET scan; MMSE: 29.09 + 0.83 points). HCs were recruited by word-of-mouth advertisement and consist mainly of spouses of patients.

### Genetic analysis

An amplicon-length analysis and repeat-primed PCR assay were used to screen for the presence of the GGGGCC hexanucleotide expansion in the first intron of C9ORF72. A repeat expansion of ≥30 was considered to be pathological^20^.

### ^18^F-fluorodeoxyglucose positron-emission tomography

Imaging was performed at the FTLD-C centers on different PET-scanners according to their respective local protocols: for an overview of which see Supplementary Table 1. To control for interscanner variance images were smoothed with a wide kernel and included nuisance variables for site and scanner type in the imaging analyses.

### Statistics

Image pre-processing included spatial normalization using Statistical Parametric Mapping (SPM8)^21^ to the PET template provided within the software by affine registration (16 nonlinear iterations, 7 × 9 × 7 basis functions), and smoothing (12 mm FWHM).

For regional voxel-based group comparisons of cerebral glucose metabolism SPM8 was used. All images were normalized to the pons as done previously^22^. Separate analysis of variance (ANOVA) group comparisons between C9+ and HC, between C9− and HC, and between C9+ and C9− were calculated, and analyses were controlled for site, scanner type, sex, age, and MMSE score.

For ROI analyses all ROI values were again referenced to the pons, which did not vary in metabolism across patients and HCs. Clusters were anatomically defined using the Automated Anatomical Labelling software package within SPM^23^.

Thalamus and cerebellar ROI receiver-operating characteristic (ROC) curves were plotted and areas under the curves (AUCs) were calculated as well as Youden index (sensitivity + specificity− 1) for best thresholds between groups and thresholds of best specificity. Spearman’s correlations between thalamic tracer uptake and clinical severity, as measured by the MMSE and CDR global scores, were calculated. A composite psychosis score as a binary indicator of psychotic symptomatology was determined by the investigators in any patient based on the occurrence of delusions and/or hallucinations (absence = 0; occurrence = 1 within the last 3 months). In addition, a more “liberal” psychosis score was created based on the occurrence of delusions and/or hallucinations and/or obsessive-compulsive behaviors and/or complex repetitive behaviors and/or unusual addiction—because these behaviors might be caused by delusional thinking. All neuropsychiatric symptoms were obtained at the patients’ visits according to clinical observation, and patient and caregiver reports. Group differences of thalamus uptake between the patients with and without presence of psychosis were calculated using Mann-Whitney *U* test.

Co-diagnosis of ALS at the time of exam was included as a variable in order to analyze its possible influence. Univariate ANOVA of thalamic tracer uptake was conducted using the variables C9+/C9− and ALS present/absent as factors.

The Statistical Package for the Social Sciences 23 was used to compute group comparisons regarding clinical symptoms and ROI metabolic differences.

A statistical threshold of <0.05 was considered as statistically significant for all ROI analyses and of <0.05 corrected for family-wise error (FWE) on cluster level for all voxel-based analyses.

## Results

Of the 22 C9+ patients 16 were diagnosed as definite (with a known pathogenic mutation) bvFTD, according to the diagnostic criteria^24^. Four patients were diagnosed as bvFTD in combination with ALS, 1 patient with semantic variant of primary progressive aphasia, and 1 patient with corticobasal degeneration with cognitive and behavioral impairment^25^. Accordingly, the C9− group was comprised of 16 patients with probable bvFTD, 4 patients with bvFTD in combination with ALS, 1 patient with semantic variant of primary progressive aphasia, and 1 patient with corticobasal degeneration with cognitive and behavioral impairment.

### Similar rate of psychosis in C9+ and C9−FTD

For demographic characteristics, neuropsychological (CERAD-NAB) test results, and the occurrence of psychotic symptoms see Table 1. Demographic variables were not significantly associated with C9ORF72 status, as seen by Chi-square and Mann-Whitney *U* values (*p*-value range: 0.494–0.925). There was no significant difference between C9+ and C9− regarding the occurrence of psychosis, irrespective if defined either strictly or liberally.

**Table 1:** Demographic characteristics, neuropsychological (CERAD-NAB) test results, and psychotic symptoms

	C9 positive	C9 negative	*p* Value
Male/female	9/13 (41%/59%)	8/14 (36%/64%)	0.757
Age at PET scan	*M* = 60.56 ± 9.96; Mdn = 63.50 (1st: 55.75, 3rd: 68.00)	*M* = 63.25 ± 9.46; Mdn = 61.50 (1st: 56.00, 3rd: 72.00)	0.925
Age at symptom onset	*M* = 57.55 ± 12.73; Mdn = 60.50 (1st: 45.75, 3rd: 68.00)	*M* = 59.36 ± 11.86; Mdn = 60.00 (1st: 53.00, 3rd: 69.50)	0.650
Disease duration from symptom onset to PET scan	*M* = 4.54 ± 4.39 Mdn = 2.53 (1st: 1.63, 3rd: 7.06)	*M* = 4.19 ± 4.62; Mdn = 2.41 (1st: 1.75, 3rd: 5.08)	*0.580*
CERAD-NAB phonematic fluency	*M* = 5.63 ± 2.28; Mdn = 5.00 (1st: 3.25, 3rd: 7.00)	*M* = 6.20 ± 4.16; Mdn = 6.00 (1st: 3.00, 3rd: 9.00)	0.952
CERAD-NAB 15-item Boston Naming Test	*M* = 12.35 ± 2.37; Mdn = 13.00 (1st: 12.00, 3rd: 14.00)	*M* = 12.23 ± 2.94; Mdn = 13.00 (1st: 11.00, 3rd: 14.25)	0.818
MMSE score	*M* = 24.32 ± 6.60; Mdn = 25.50 (1st: 25.00, 3rd: 28.00)	*M* = 23.23 ± 6.04; Mdn = 25.00 (1st: 21.50, 3rd: 27.00)	0.494
CERAD-NAB word list learning	*M* = 15.53 ± 3.97; Mdn = 16.00 (1st: 12.00, 3rd: 17.00)	*M* = 13.50 ± 4.64; Mdn = 12.50 (1st: 10.00, 3rd: 16.00)	0.104
CERAD-NAB delayed verbal recall	*M* = 5.68 ± 5.27; Mdn = 5.00 (1st: 3.00, 3rd: 7.00)	*M* = 3.37 ± 2.11; Mdn = 3.00 (1st: 2.00, 3rd: 5.00)	0.073
CERAD-NAB visuoconstruction	*M* = 9.60 ± 1.57; Mdn = 10.00 (1st: 8.00, 3rd: 11.00)	*M* = 9.15 ± 2.48; Mdn = 10.00 (1st: 7.50, 3rd: 11.00)	0.841
CERAD-NAB delayed non-verbal recall	*M* = 5.65 ± 2.72; Mdn = 6.00 (1st: 3.00, 3rd: 7.75)	*M* = 6.05 ± 3.95; Mdn = 5.00 (1st: 3.00, 3rd: 10.00)	0.627
CDR sum of boxes score	*M* = 5.14 ± 4.41; Mdn = 3.25 (1st: 2.50, 3rd: 7.25)	*M* = 5.23 ± 2.99; Mdn = 4.75 (1st: 3.00, 3rd: 6.25)	0.569
Hallucinations and/or delusions present	14%	19%	0.680
Psychotic symptoms (liberal definition) present	32%	45%	0.353

Mean ± standard deviation; median (1st and 3rd quartiles)

*M* mean, *Mdn* median, *1st and 3rd* first and third quartiles of ranked dataset, *CDR* Clinical Dementia Rating, *MMSE* Mini-Mental State Examination, *PET* positron-emission tomography, *CERAD-NAB* Consortium to Establish a Registry of Alzheimer’s Disease-Neuropsychological Assessment Battery

### Voxel-based comparisons show a glucose hypometabolism in both thalami in C9+ as compared to C9−

**Fig. 1:**
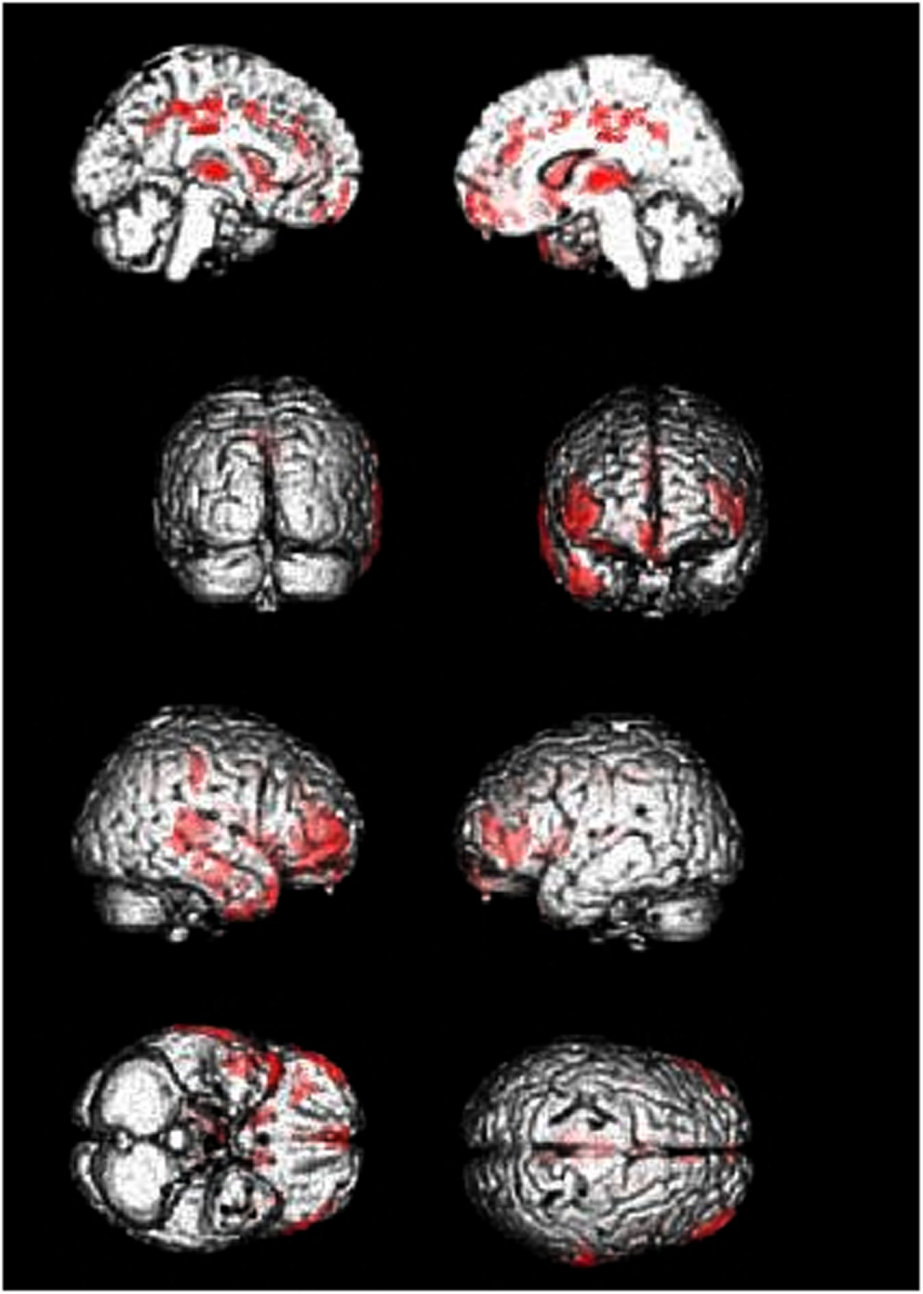
C9+ patients compared with controls. C9+ < healthy control; voxel-based comparison, *p* < 0.05 family-wise error corrected; clusters rendered to a three-dimensional brain within Statistical Parametric Mapping, views from mesial left, mesial right, back, front, lateral right, lateral left, bottom, above

Cerebral glucose metabolism as measured with 18-FDG-PET was compared between patient groups and HCs using regional voxel-based group comparisons. As compared to HC 12 significant hypometabolic clusters were detected in C9+ patients comprising right and left frontal medial orbital gyrus, right gyrus rectus, right medial frontal lobe, right middle orbital gyrus, right and left frontal inferior triangular gyrus, left medial frontal lobe, right medial and superior temporal gyrus, right middle temporal pole, right inferior temporal lobe, right insula and right Rolandic operculum, right anterior cingulate gyrus, right and left middle cingulate gyrus, the right and left thalamus, and right and left caudate nuclei (Fig. 1). Local maxima are provided in Supplementary Table 2.

**Fig. 2:**
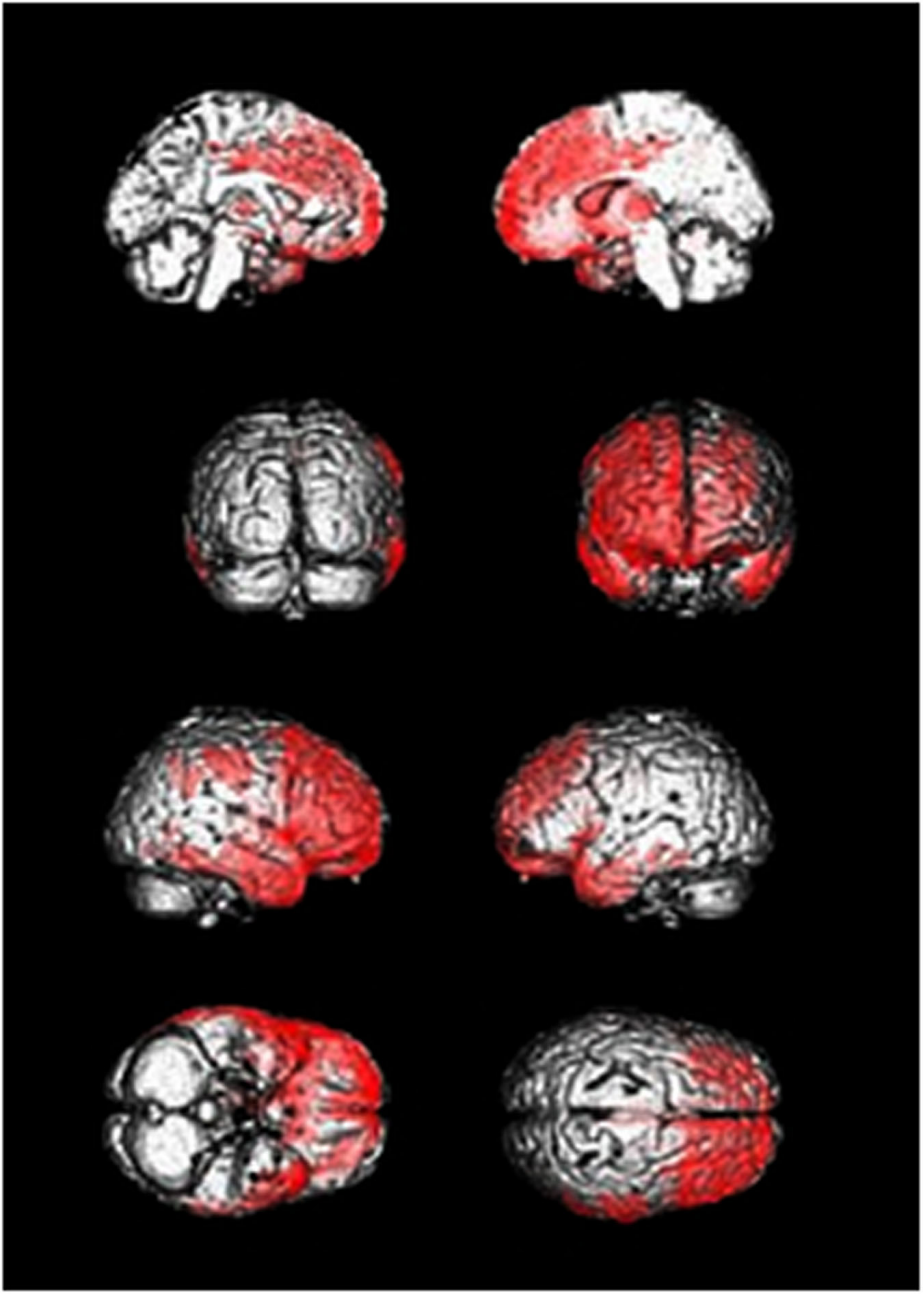
C9- patients compared with controls. C9− < healthy control; voxel based comparison, *p* < 0.05 family-wise error corrected; clusters rendered to a three-dimensional brain within Statistical Parametric Mapping; views from mesial left, mesial right, back, front, lateral right, lateral left, bottom, above

As compared to HCs two significant hypometabolic clusters were found in C9− comprising the right and left middle and superior frontal gyri, right inferior frontal gyrus, right supplementary motor area, right and left inferior temporal gyri, right and left temporal poles, right middle and superior temporal gyri, left inferior and medial temporal gyri, right supramarginal gyrus, right insula, right cingulate gyrus, right thalamus, right caudate nucleus, right postcentral gyrus, and right inferior parietal lobule, as shown in Fig. 2. Local maxima are provided in Supplementary Table 3.

**Fig. 3:**
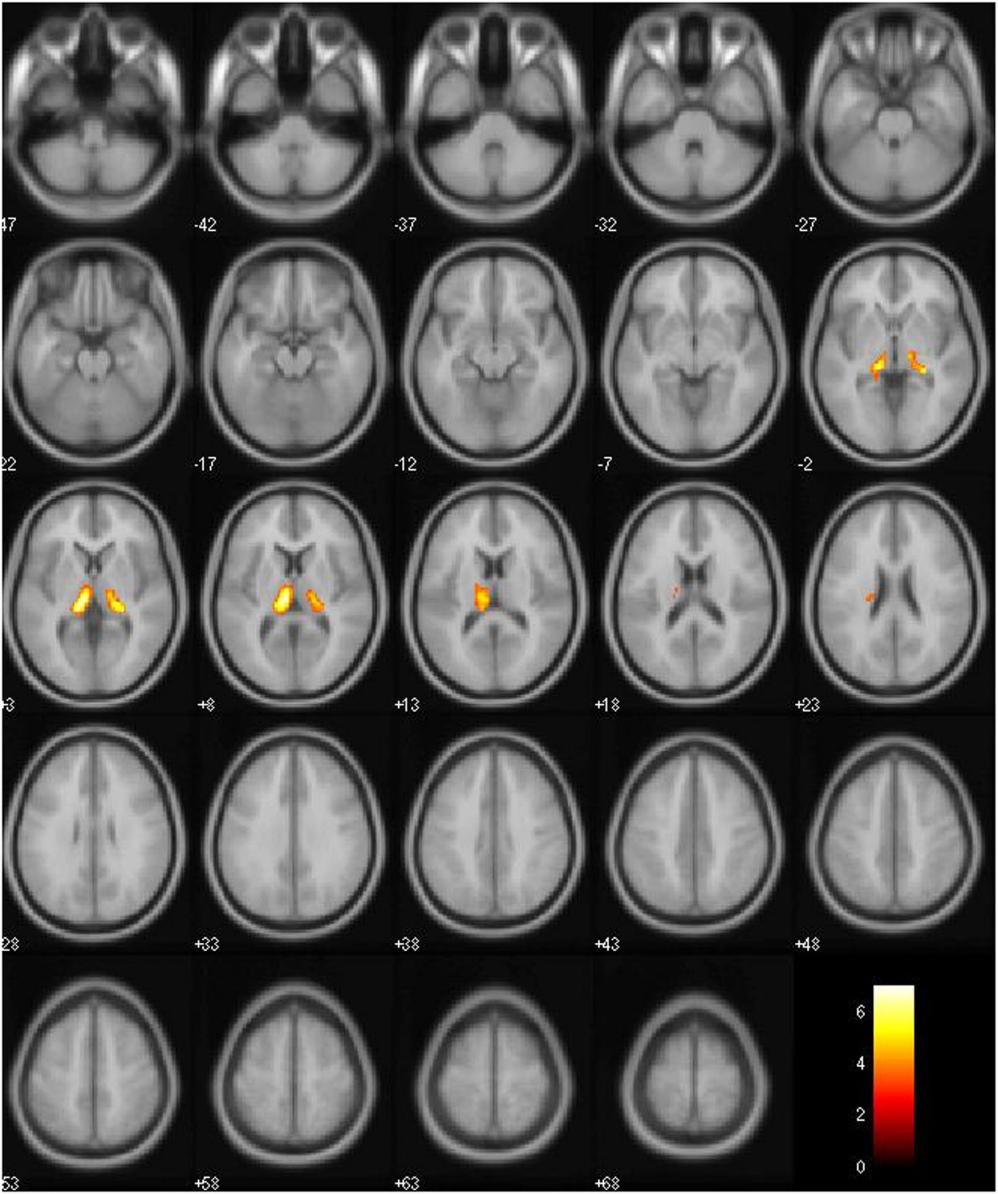
C9+ patients compared with C9− patients. Clusters overlaid on a mean of 152 T1 MRIs within Statistical Parametric Mapping; each slice is separated by 5 mm

Comparison of C9+ and C9− yielded two significant hypometabolic clusters in the left and right thalamus for C9+ (Fig. 3); local maxima are provided in Supplementary Table 4. Removing scans of patients with the outlier diagnoses semantic variant of primary progressive aphasia and corticobasal degeneration from the analysis did not substantially change the results (data not shown).

No significant hypermetabolic clusters were observed in C9+ compared to C9−.

### ROI-based analyses show diagnostic value of FDG-PET but no correlation between thalamic metabolism and clinical features

#### Thalamus

**Fig. 4:**
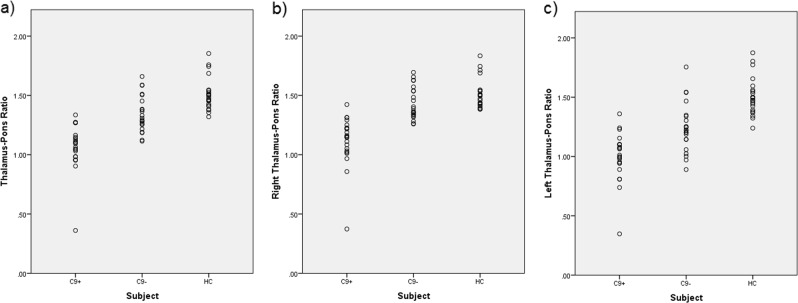
Thalamus-Pons FDG uptake ratios for patients and controls. **a** Global thalamic ^18^F-fluorodeoxyglucose (FDG) uptake in C9+, C9−, and healthy controls (HC): scatterplots. **b** Right thalamic FDG uptake in C9+, C9−, and HC: scatterplots. **c** Left thalamic FDG uptake in C9+, C9−, and HC: scatterplots

Global as well as left and right thalamic tracer uptake differed significantly between all three groups, C9 + < C9− < HC (*p* < 0.01 for each comparison). Thalamus/pons FDG uptake ratios for patient and control groups are provided in Supplementary Table 5. Scatterplots are provided in Fig. 4a–c and boxplots in Supplementary Fig. 1.

**Fig. 5:**
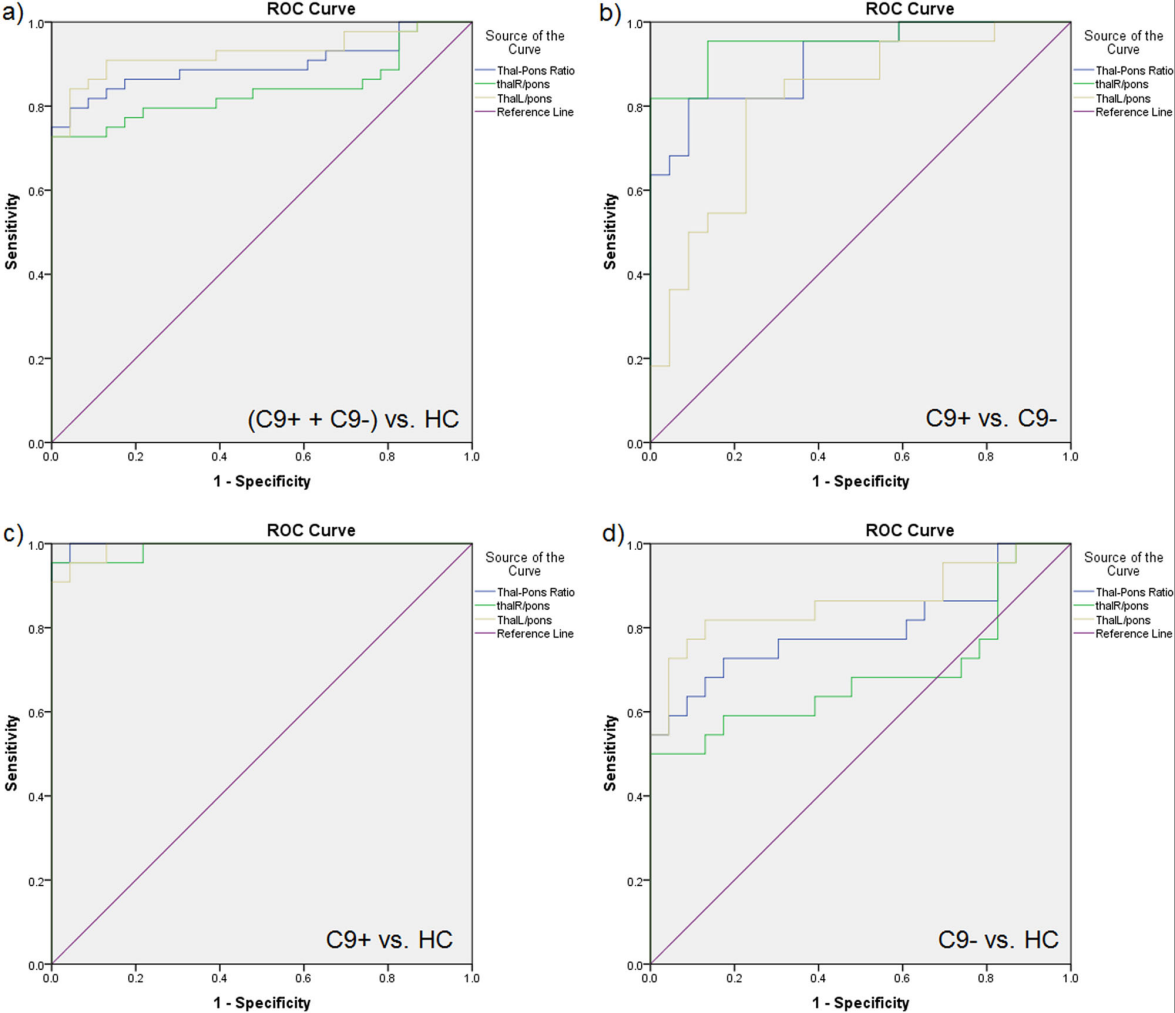
ROC curve analyses. Receiver-operating characteristic (ROC) curve plots for global and hemispheric thalamus/pons ratios including reference line; a whole patient group (C9+ and C9−) vs. healthy controls (HCs); **b** C9+ vs. C9−; **c** C9+ vs. HC; **d** C9– vs. HC. ThalR right thalamus, ThalL left thalamus

To test the diagnostic relevance of thalamic hypometabolism we performed ROC curve analyses. ROC curves between patients and HC and between patient groups are provided in Fig. 5. AUCs, Youden’s Index, and 100% specificity cutoffs with corresponding cutoff value are shown in Supplementary Table 6. All AUCs were statistically significant with *p*-values < 0.001.

Neither global nor right or left thalamic FDG uptake seen in the whole patient group and in C9 subgroups significantly correlated with disease severity, as measured by CDR (*p*-value range: 0.425–0.978) and MMSE (*p*-value range: 0.070–0.981) or disease duration since onset (*p*-value range: 0.458–0.697).

Thalamic tracer uptake did not significantly differ between patients with and without psychotic symptoms (*p* = 0.638). A Chi-square test revealed a lack of association between *C9ORF72* status and presence of “psychosis”, *X*^2^(1) = 0.863, *p* = 0.353 (Cramer’s *V* = 0.140, *p* = 0.353).

Since abovementioned studies in ALS patients with C9 mutation had described a lower thalamic glucose uptake in C9+ as compared to C9−, we added an ANOVA of thalamic FDG uptake using diagnosis of FTD-ALS and *C9ORF72* status as factors. No main effect of a diagnosis of FTD-ALS, (*F*(1) = 1.610, *p* = 0.212, *ηp*^2^ = 0.039) but of *C9ORF72* status (*F*(1) = 40.611, *p* < 0.001, *ηp*^2^ = 0.504) and an ordinal interaction effect between *C9ORF72* status and a diagnosis of FTD-ALS (*F*(1) = 8.524, *p* = 0.006, *ηp*^2^ = 0.176) was found. Accordingly, a coexistent diagnosis of ALS does not explain differences in thalamic FDG uptake but the effect of C9ORF72 status on FDG uptake is modified by the presence of FTD-ALS: in patients with concomitant ALS *C9ORF72* status resulted in accentuated significant thalamic FDG uptake difference (*F* = 26.384, *p* < 0.001) as compared to patients without (*F* = 16.394, *p* < 0.001).

### Cerebellum

Cerebellar FDG uptake for global or for right and left regions was not significantly different across groups (*p*-value range: 0.089–0.892) as shown in Supplementary Table 7. Notably, a few patients showed higher FDG uptake than HCs as shown in Supplementary Fig. 2. Although the variance of global cerebellar uptake was higher in C9+ as compared to C9−, differences in variance were not statistically significant (*p* = 0.130). Neither in the entire patient group nor in the genetic subgroups did cerebellar uptake correlate with disease severity (measured with CDR and MMSE) or duration of disease since onset (*p*-value range: 0.172–0.999).

## Discussion

The present FDG-PET study compared cerebral glucose metabolism of C9FTLD patients with matched non-carriers and with HCs. Voxel-based comparisons revealed a significant hypometabolic pattern in mutation carriers relative to HCs across widespread frontal and temporal areas, cingulate cortex, Rolandic operculum, caudate nuclei, and thalami. As compared to HCs, C9− showed a significant hypometabolism similar to C9+. The comparison between both FTLD groups revealed a significant reduction of metabolism in both thalami in C9+.

ROI analyses showed that both carriers and non-carriers had a significantly lower thalamus/pons glucose uptake ratio than HCs; C9+ also had a significantly lower thalamus/pons glucose uptake ratio when compared to C9−. Uptake ratios did not correlate with disease severity.

ROC curve analysis revealed that thalamus/pons uptake ratios could separate the whole group of patients from HCs with an AUC of 0.897 for the bilateral thalami. The ROC curve analyses also distinguished carriers from non-carriers with an AUC of 0.909, making FDG thalamic uptake a favorable biomarker for the detection of FTLD and particularly for the detection of C9FTLD.

To our knowledge, this is the first study that reports glucose metabolic patterns on a group basis within a cohort of C9FTLD patients and it provides valuable insights into brain function in C9FTLD. The findings of our study corroborate the results of a wealth of structural imaging studies, e.g. refs. ^26–28^, connectivity analyses^13^ and neuropathological studies^10,29^ that identified the thalamus as a key region in C9FTLD. It has already been shown that the thalamus is affected in sporadic FTLD^10^ as well as in other neurodegenerative diseases such as Alzheimer’s disease^30^. However, the striking difference in glucose uptake between C9+ and C9− patients observed in our study warrants the conclusion that the thalamus is particularly vulnerable in C9FTLD. This is consistent with the results of recent MRI studies that describe atrophy of the thalamus even in presymptomatic C9ORF72 mutation carriers^31^.

The thalamus acts as a hub, relaying information between different subcortical areas and the cerebral cortex. Dorsal thalamic nuclei are part of the three frontal-subcortical circuits. Yokoyama et al. have already hypothesized that the thalamus may be uniquely affected in C9ORF72 expansion carriers, potentially representing the “epicenter of vulnerability”^32^. The degeneration of the dorsal thalamic nuclei network in C9FTLD might result in a disruption of the frontal-subcortical circuits and consequently in a rather diffuse (as compared to other neurodegenerative diseases) pattern of cortical atrophy. Deterioration of these frontal-subcortical circuits leads to a variety of neuropsychiatric symptoms.

The alterations of thalamic vulnerability in C9FTLD might be partially explained by the unique neuropathology. Neuropathological studies identified both DPR and TDP-43 pathology in the thalamus in most C9FTLD cases^33,34^. However, a case with C9FTD was reported with the medial pulvinar nucleus of the thalamus standing out as a region of prominent neuronal atrophy, widespread DPR pathology, but nearly absent TDP-43 pathology. Therefore, it is unclear whether C9orf72-associated neurodegeneration can occur in the absence of pathological TDP-43^35^.

Contrary to other studies^6,7^, we did not detect statistically significant differences regarding the prevalence of psychotic symptoms between C9+ and C9− patients in our cohort. This is surprising and might in part be explained by the fact that the majority of patients were recruited from psychiatric hospitals. Thus, patients with prominent neuropsychiatric symptoms were more likely to be included.

Thalamus uptake did not differ in patients with or without “psychosis” (liberally defined), suggesting that a functional deficit of the thalamus is not associated with psychotic symptoms in FTLD.

A recent MRI study observed that increased psychotic symptoms in C9ORFf72 expansion carriers correlated with atrophy in a distributed cortical and subcortical network including discrete regions of frontal, temporal, and occipital cortices as well as thalamus, striatum, and cerebellum^6^. This network is comparable to a circuit known to be affected in schizophrenia^36^

In the present study, no significant metabolic reduction was detected in the cerebellum across carriers and non-carriers. This result appears to contradict the findings of several MRI studies that observed cerebellar atrophy in early and even in presymptomatic disease stages of C9FTLD^31,37^. In addition, a recent study had confirmed cerebellar neuron loss using immunohistochemical staining^38^.

Some limitations of the present study need to be considered. First, the patient cohort—despite being the largest C9FTLD FDG cohort reported so far—is rather small and clinically heterogeneous. Second, as a multicenter study, six different sites provided clinical data and PET scans that were acquired on different scanners using varying protocols. These dissimilarities required rigorous control, potentially enabling the failure to detect subtle differences among the patient groups. A third limitation of the study is that although MRIs were available for all patients for diagnostic purposes, only a minority had got MRI following a standardized protocol that would allow comparison between centers. Therefore, neither a comparison between FDG-PET measures and structural MRI measures nor an atrophy correction can be provided.

Furthermore, a considerable disadvantage of FDG-PET is that spatial resolution is lower than with MRI, thus preventing analyses of thalamic substructures.

Finally, the spatial normalization step, using the SPM PET template image, instead of producing a template specific to the cohort and the tracer used in this study, might be considered suboptimal^39^. However, the SPM-derived results correspond well to the ROI-derived results, so no major bias is suspected.

### Future directions

Future studies in large cohorts of patients and presymptomatic mutation carriers are needed to further elucidate the key role of the thalamus in C9FTLD. In vitro approaches are necessary to investigate how C9ORF72 expansions increase the vulnerability of the thalamus. Multimodal neuroimaging studies are required to provide further information specifying the role of the thalamus at disease onset and progression as well as within the cerebello-thalamo-cortical circuits. While thalamic FDG uptake differed excellently between C9+, C9−, and HC, the question of whether thalamic structure or function is suitable as a biomarker for differential diagnosis, disease progression and therapy response in (C9)FTLD requires further investigation. Large neuropathological approaches need to analyze tissue from the thalamus together with tissue from the cortico-subcortical circuits in in order to identify the foci earliest affected and the chronological order of protein pathology.

## Supplementary information

TP Supplementary Information
