# Supplementary material for: FDG-PET underscores the key role of the thalamus in frontotemporal lobar degeneration caused by C9ORF72 mutations
Source: Transl Psychiatry. 2019 Jan 31;9:54. doi: 10.1038/s41398-019-0381-1 (PMC6355852; doi:10.1038/s41398-019-0381-1)
Supplement: Supplementary file 1 — TP Supplementary Information [file 41398_2019_381_MOESM1_ESM.doc]

**Supplementary Information**

**Supplementary Table 1:** Overview: Scanners and local standards

| **Study Site** | **Scanner model, local standards** |
| --- | --- |
| Bonn/ Cologne | Scanner type: Siemens Biograph 2  Procedure as already published in {Varrone, 2009 #9337}.  Scanner type: Siemens Biograph mCT Flow 128 Edge  Activity: ~185 MBq [18F]  Incubation time: 30 minutes  Emission time: 10 minutes  Acquisition mode: 3D  Axial field of view: 216 mm  Attenuation correction: low-dose CT  Reconstruction: 3-D OSEM algorithm (four iterations, 12 subsets)  Gaussian filter: 5 mm full width at half maximum (FWHM)  Image matrix: 400x400  Slices: 148, slice thickness of 1.5 mm |
| Homburg | Scanner type: Siemens Biograph 40 mCT  Activity: 180 MBq  Incubation time: 45 minutes  Emission time: 10 minutes  Axial field of view 208 mm  Attenuation correction: low-dose CT (100 mAs, 120 kV)  Reconstruction: List mode (3 iterations, 24 subsets)  Image matrix: 128x128 and 400x400  Slices: 40, slice thickness of 3 mm |
| Munich (Technical University) | Scanner type: Siemens Biograph mMR  Scanner type: Siemens SOMATOM Definition AS mCT  Scanner type: Siemens Biograph 40 mCT  Scanner type: Siemens Biograph128 mCT  Scanner type: Siemens ECAT EXACT HR+ PET  Procedure as already published in {Diehl-Schmid, 2007 #7755}; {Perneczky, 2007 #7595} {Titov, 2017 #9304} |
| Munich (Ludwig-Maximilians- University) | GE Discovery PET/CT  Siemens ECAT EXACT HR+ PET  Procedure as already published in {Brendel, 2017 #9336} |
| Rostock | Scanner type: Phillips Gemini TF PET/CT  Activity: ~150 MBq  Incubation time: 30 minutes  Emission time: 20 min  Acquisition mode: 3D  Axial field of view: 254 mm  Attenuation correction: low-dose CT  Reconstruction: 3D RAMLA algorithm  Gaussian filter: 4.7 mm full width at half maximum (FWHM)  Image matrix: 128x128  Slices: 90 |
| Ulm | Scanner type: Siemens Biograph 40 mCT  Activity: ~150 MBq  Incubation time: 30 minutes  Emission time: 20 min  Acquisition mode: 3D  Reconstruction: 3D OSEM algorithm  Gaussian filter: 5.0 mm full width at half maximum (FWHM)  Image matrix: 400x400  Slices: 148, slice thickness of 3 mm |

**Legend to Supplementary Table 1:** Local standards; site and scanner information for patients collected from various cities within Germany**.**

**Supplementary Table 2:** C9+ hypometabolism as compared to HC: local maxima

[maxima C9+ < HC]

| Voxel Cluster Amount (*k*_E_) | X,Y,Z Coordinates | *p*-value |
| --- | --- | --- |
| 817 | -6, -24, 32 | <0.001 |
| 740 | 8, -14, 6 | <0.001 |
| 663 | 44, 54, 4 | <0.001 |
| 466 | 66, -18, -24 | <0.001 |
| 422 | 48, 20, -28 | <0.001 |
| 407 | 22, -8, 20 | <0.001 |
| 357 | -48, 42, 4 | <0.001 |
| 306 | 6, 62, -12 | <0.001 |
| 241 | -12, 10, 8 | 0.003 |
| 225 | 6, 36, 22 | 0.004 |
| 201 | 50, 2, 6 | 0.008 |
| 140 | 4, 6, 44 | 0.048 |

**Legend to Supplementary Table 2:** Local maxima, p<0.05 FWE cluster corrected. Coordinates reflect Talairach space**.**

**Supplementary Table 3:** C9- hypometabolism as compared to HC: local maxima

[maxima C9- < HC]

| Voxel Cluster Amount (*k*_E_) | X,Y,Z Coordinates | *p*-value |
| --- | --- | --- |
| 225890 | 10, 26, 32 | <0.001 |
| 724 | 56, -34, 52 | <0.001 |

**Legend to Supplementary Table 3:** Local maxima, p<0.05 FWE cluster corrected. Coordinates reflect Talairach space**.**

**Supplementary Table 4:** C9+ hypometabolism as compared to C9-: local maxima

[maxima C9+ < C9-]

| Voxel Cluster Amount (*k*_E_) | X,Y,Z Coordinates | *p*-value |
| --- | --- | --- |
| 709 | -8, -20, 4 | <0.001 |
| 364 | 14, -18, 2 | 0.013 |

**Legend to Supplementary Table 4:** Local maxima, p<0.05 FWE cluster corrected. Coordinates reflect Talairach space**.**

**Supplementary Table 5**: Thalamus/pons FDG tracer uptake across groups

|  | C9+ | C9- | HC | C9+ vs. C9- | C9+ vs. HC | C9- vs. HC | Patient (C9+,-) vs. HC |
| --- | --- | --- | --- | --- | --- | --- | --- |
| Global Thalamus | 1.06 ± 0.20;  Mdn=1.08 (1st: 0.98  3^rd^: 1.15) | 1.34 ± 0.15; Mdn= 1.30,  (1^st^: 1.25  3^rd^: 1.47) | 1.51 ± 0.14; Mdn=1.48 (1^st^: 1.41  3^rd^: 1.54) | *p* < 0.001 | *p* < 0.001 | *p* < 0.001 | *p* < 0.001 |
| Right Thalamus | 1.12 ± 0.21; Mdn=1.15  (1^st^: 1.03  3^rd^: 1.23) | 1.43 ± 0.14; Mdn= 1.38  (1^st^: 1.33  3^rd^: 1.55) | 1.51 ± 0.12; Mdn=1.50  (1^st^: 1.42  3^rd^: 1.54) | *p* < 0.001 | *p* < 0.001 | *p* < 0.001 | *p* < 0.001 |
| Left Thalamus | 1.0 ± 0.21; Mdn=1.0 (1^st^: 0.93  3^rd^: 1.10) | 1.24 ± 0.21; Mdn=1.22  (1^st^: 1.12  3^rd^: 1.34) | 1.51 ± 0.16; Mdn=1.48  (1^st^: 1.39  3^rd^: 1.56) | *p* < 0.001 | *p* < 0.001 | *p* < 0.001 | *p* < 0.001 |

**Legend to Supplementary Table 5**: Thalamus/pons FDG uptake ratios for patient and control groups. p-values: Mann-Whitney U tests. Mean ± standard deviation; median (1st and 3rd quartiles). M = mean, Mdn = Median, 1st and 3rd = first and third quartiles of ranked dataset

**Supplementary Table 6:** Thalamic FDG uptake ROC curve analysis: AUCs, Youden’s Index and 100% specificity cut-offs (with corresponding cut-off value)

|  | Global thalamus | Right thalamus | Left thalamus |
| --- | --- | --- | --- |
| Whole Patient Group v. HC | AUC: 0.897  J: 0.731 (1.37)  Spec: 100% (1.31) | AUC: 0.839  J: 0.578 (1.42);  Spec: 100% (1.37) | AUC: 0.927  J: 0.779 (1.36)  Spec: 100% (1.23) |
| C9+ vs. C9- | AUC: 0.909  J: 0.727 (1.17);  Spec: 100% (1.11) | AUC: 0.955  J: 0.819 (1.32)  Spec: 100% (1.26) | AUC: 0.810  J: 0.591 (1.12)  Spec: 100% (0.85) |
| C9+ vs. HC | AUC: 0.998  J: 0.957 (1.34)  Spec: 100% (1.30) | AUC: 0.990  J: 0.955 (1.35)  Spec: 100% (1.35) | AUC: 0.992  J: 0.912 (1.28)  Spec: 100% (1.23) |
| C9- vs. HC | AUC: 0.796  J: 0.553 (1.40)  Spec: 100% (1.31) | AUC: 0.688  J: 0.417 (1.41)  Spec: 100% (1.38) | AUC: 0.862  J: 0.688 (1.36)  Spec: 100% (1.23) |

**Legend to Supplementary Table 6:** All AUCs statistically significant with p-values < 0.001; AUC = Area under the curve; J = Youden's Index; Spec:100% = cut-off for 100%

**Supplementary Table 7:** Cerebellum/pons FDG tracer uptake across groups

|  | C9+ | C9- | HC | C9+ vs. C9- | C9+ vs. HC | C9- vs. HC |
| --- | --- | --- | --- | --- | --- | --- |
| Global Cerebellum | 1.37 ± 0.30; Mdn=1.32  (1^st^: 1.25,  3^rd^: 1.44) | 1.37 ± 0.16;  Mdn=1.36  (1^st^: 1.28  3^rd^: 1.45) | 1.33 ± 0.058; Mdn=1.34  (1^st^: 1.28  3^rd^: 1.37) | *p*=0.496 | *p*=0.820 | *p*= 0.220 |
| Right Cerebellum | 1.36 ± 0.29;  Mdn=1.30  (1^st^: 1.25  3^rd^ : 1.46) | 1.35 ± 0.17; Mdn=1.35  (1^st^: 1.27  3^rd^ : 1.43) | 1.33 ± 0.056; Mdn=1.34  (1^st^: 1.27  3^rd^ : 1.36) | *p*=0.639 | *p*=0.733 | *p*=0.454 |
| Left Cerebellum | 1.38 ± 0.31;  Mdn=1.30  (1^st^: 1.26  3^rd^ : 1.45) | 1.38 ± 0.15;  Mdn=1.37  (1^st^: 1.30  3^rd^ : 1.46) | 1.33 ± 0.064;  Mdn=1.34  1^st^: 1.28  3^rd^ : 1.37) | *p*=0.324 | *p*=0.892 | *p*=0.089 |

**Legend to Supplementary Table 7:** cerebellum/pons FDG uptake ratios for patient and control groups. p-values: Mann-Whitney U tests. Mean ± standard deviation; median (1st and 3rd quartiles). M = mean, Mdn = Median, 1st and 3rd = first and third quartiles of ranked dataset

**Supplementary Figure 1:** Thalamic FDG uptake: boxplots


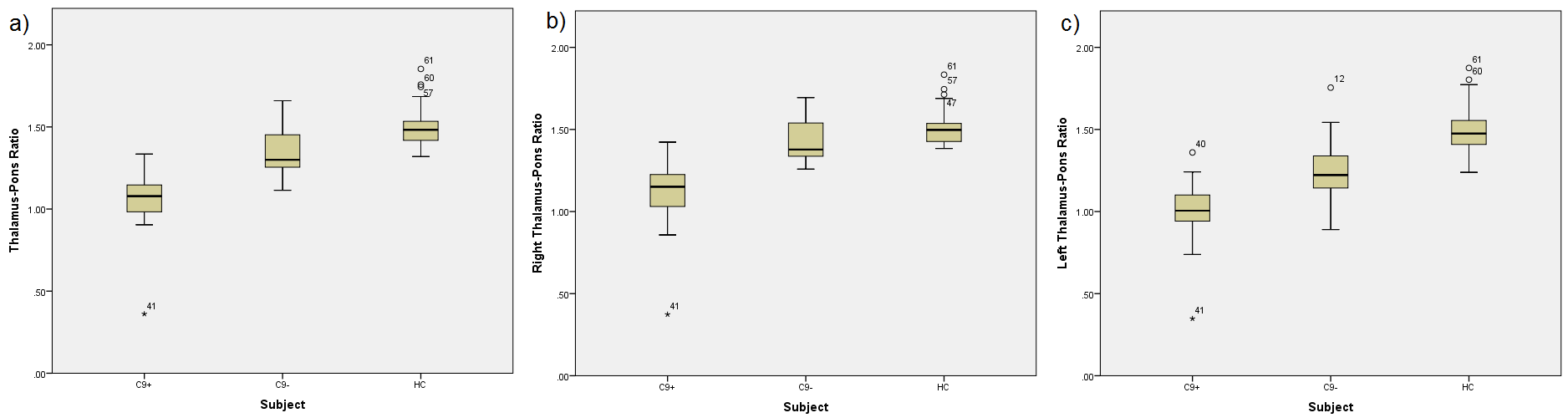


**Legend to Supplementary Figure 1:** Thalamus/pons FDG uptake ratios. A) global, b) right, c) left thalamus

**Supplementary Figure 2:** Cerebellar FDG uptake: boxplots


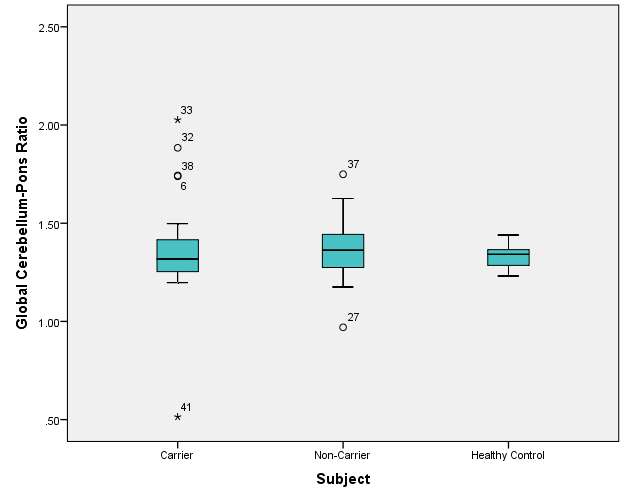


**Legend to Supplementary Figure 2:** Global cerebellum/pons FDG uptake ratios.
